# Looking through the imaging perspective: the importance of imaging necrosis in glioma diagnosis and prognostic prediction – single centre experience

**DOI:** 10.2478/raon-2024-0014

**Published:** 2024-02-21

**Authors:** Hui Ma, Shanmei Zeng, Dingxiang Xie, Wenting Zeng, Yingqian Huang, Liwei Mazu, Nengjin Zhu, Zhiyun Yang, Jianping Chu, Jing Zhao

**Affiliations:** Department of Radiology, The First Affiliated Hospital, Sun Yat-sen University, Guangzhou, Guangdong Province, China

**Keywords:** glioma, necrosis, MRI, molecular markers, prognosis

## Abstract

**Background:**

The aim of the study was to investigate the diagnostic value of imaging necrosis (Im_necrosis_) in grading, predict the genotype and prognosis of gliomas, and further assess tumor necrosis by dynamic contrast-enhanced MR perfusion imaging (DCE-MRI).

**Patients and methods:**

We retrospectively included 150 patients (104 males, mean age: 46 years old) pathologically proved as adult diffuse gliomas and all diagnosis was based on the 2021 WHO central nervous system (CNS) classification. The pathological necrosis (Pa_necrosis_) and gene mutation information were collected. All patients underwent conventional and DCE-MRI examinations and had been followed until May 31, 2021. The Im_necrosis_ was determined by two experienced neuroradiologists. DCE-MRI derived metric maps have been post-processed, and the mean value of each metric in the tumor parenchyma, peritumoral and contralateral area were recorded.

**Results:**

There was a strong degree of inter-observer agreement in defining Im_necrosis_ (Kappa = 0.668, p < 0.001) and a strong degree of agreement between Im_necrosis_ and Pa_necrosis_ (Kappa = 0.767, p < 0.001). Compared to low-grade gliomas, high-grade gliomas had more Im_necrosis_ (85.37%, p < 0.001), and Im_necrosis_ significantly increased with the grade of gliomas increasing. And Im_necrosis_ was significantly more identified in *IDH*-wildtype, *1p19q*-non-codeletion, and *CDKN2A/B*-homozygous-deletion gliomas. Using multivariate Cox regression analysis, Im_necrosis_ was an independent and unfavorable prognosis factor (Hazard Ratio = 2.113, p = 0.046) in gliomas. Additionally, extravascular extracellular volume fraction (*ve*) in tumor parenchyma derived from DCE-MRI demonstrated the highest diagnostic efficiency in identifying Pa_necrosis_ and Im_necrosis_ with high specificity (83.3% and 91.9%, respectively).

**Conclusions:**

Im_necrosis_ can provide supplementary evidence beyond Pa_necrosis_ in grading, predicting the genotype and prognosis of gliomas, and *ve* in tumor parenchyma can help to predict tumor necrosis with high specificity.

## Introduction

Necrosis is a common feature of human cancer and is often related to a poor prognosis, especially in glioblastomas.^1,2,3^ Though the importance of necrosis in gliomas has already been addressed, necrosis was first incorporated into the determinant of the diagnosis for glioma grade in the fifth edition of the 2021 WHO classification of Tumors of the central nervous system (CNS), which highlighted and underlined the significant value of necrosis in the diagnosis and prognosis of adult diffuse gliomas.^4^ According to the latest classification, once histological necrosis is identified, a diagnosis of WHO grade 4 astrocytoma or glioblastoma is suggested. However, there is a diagnostic dilemma in grading gliomas by identifying necrosis.

Presently, necrosis is primarily determined by pathological examination, in which partial tumor specimens from specific sites of tumors obtained by surgery or biopsy at a single point in time are generally inspected.^5^ However, due to tumor heterogeneity and incompleteness of the pathological sample, some pathological necrosis is likely to be missed, which may result in an underestimation of tumor grades, especially when the molecular analysis is not available. As tumor grades influence therapeutic decisions and prognosis, it is imperative to make up for the problem of a missed diagnosis of necrosis on pathological evaluation.

Magnetic resonance imaging (MRI) is utilized for routine, noninvasive, preoperative examination in diagnosing gliomas. Pathological necrosis usually has corresponding imaging features.^6,7^ Imaging necrosis has been defined as a region within the tumor that does not enhance or shows markedly diminished enhancement, high signal intensity on T2WI, low signal intensity on T1WI, and an irregular border.^6^ Hence necrosis in gliomas, when substantially present, can be detected by conventional MRI and plays a vital role in diagnosing gliomas and predicting prognosis.^6,8,9,10,11,12,13^ Moreover, conventional and advanced MRI can acquire comprehensive morphological and pathophysiological images of entire tumors, which is impossible with pathological examinations.

Taking all of this into account, we speculated whether necrosis diagnosed by MRI (hereafter termed “imaging necrosis”, abbreviated as Im_necrosis_) could be used as a correction or a supplement to necrosis diagnosed by pathological evaluation (hereafter termed “pathological necrosis”, abbreviated as Pa_necrosis_), especially when there is no evidence of Pa_necrosis_ owing to limited sampling sites and sampling amounts. Consequently, herein, we retrospectively reviewed MRI findings of adult diffuse gliomas that were diagnosed based on the 2021 WHO CNS classification and assessed the role of Im_necrosis_ in grading, predicting the genotype and prognosis of gliomas. We also attempted to analyse tumor necrosis by dynamic contrastenhanced MR perfusion imaging (DCE-MRI) to validate quantitative imaging markers for probing tumor necrosis.

## Patients and methods

### Study participants

Patients with a primary diagnosis of glioma (June 2013–May 2021) were retrospectively included. Inclusion and exclusion criteria are presented in Supplementary Figure 1. Clinical information of patients was retrieved from the electronic medical records, and follow-up information was obtained through clinical interviews. Follow-up survival data were available until May 31, 2021. Overall survival (OS) was calculated from the initial surgery date to the date of death, or the date of the last follow-up visit if the patient was alive or lost to follow-up.

This retrospective analysis was in accordance with the ethical standards of the institutional and national research committee and was approved by the ethics committee of our institution ([2021]209). The requirement for written informed consent was waived due to the retrospective nature of this study.

### MRI parameters

Participants underwent conventional (T1/T2-weighted images [T1WI/T2WI], T2-weighted fluidattenuated inversion recovery [T2WI-FLAIR] and sagittal view of contrast-enhanced three-dimensional T1 MPRAGE images) and DCE-MRI imaging using a 3.0T MR system (Magnetom Verio, Siemens Medical Solutions, Erlangen, Germany) with a 64-channel head-neck coil. The parameter details of the conventional MRI and the DCE-MRI were elaborated in Supplementary Appendix 1.

### Image processing

**FIGURE 1.**
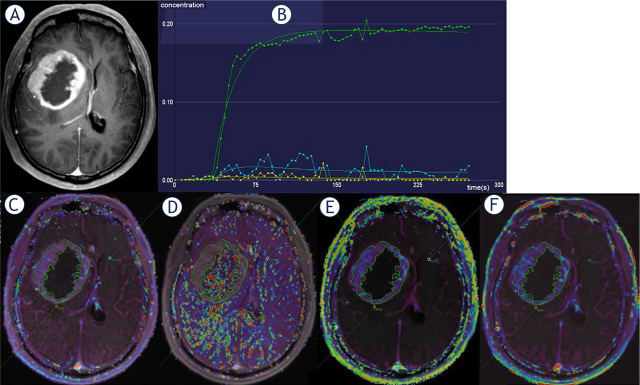
Representative ROI delineations. A 53-year-old man was diagnosed with glioblastoma, IDH-wildtype. **(A)** contrastenhanced T1-weighted image (T1WI-CE); **(B)** the time-signal intensity curve; **(C)** the transfer constant (*ktrans*) image; **(D)** rate constant (*kep*) image; **(E)** extravascular extracellular volume fraction (*ve*) image; **(F)** initial area under the curve in the first 60 s (*iauc*) image. On images, B-F, ROI 1 marked green represented tumor parenchyma, ROI 2 marked yellow represented the peripheral zones, and ROI 3 marked blue-turquoise represented contralateral normal-appearing brain tissues.

All DCE-MRI data were transferred to the post-processing workstation (detailed in Supplementary Appendix 2). Pharmacokinetic parameters, including the transfer constant (*ktrans*), extravascular extracellular volume fraction (*ve*), rate constant (*kep* = *ktrans*/*ve*), and initial area under the curve in the first 60 s (*iauc*), were automatically generated. Regions of interest (ROIs) were selected across three consecutive maximum tumor parenchyma slices. At each slice, one ROI was put in tumor parenchyma (hereafter termed “tumor”), according to T2WI-FLAIR, and enhanced T1WI, avoiding necrosis, cystic, and vessel areas. Another two approximate 2-mm-diameter ROIs were put in tumor peripheral zones (hereafter termed “edema”, within a 1-cm distance from the outer enhancing tumor margin) and contralateral normal-appearing brain tissues (hereafter termed “control”) (Figure 1). The mean values of each DCE-MRI metric was recorded.

**FIGURE 2.**
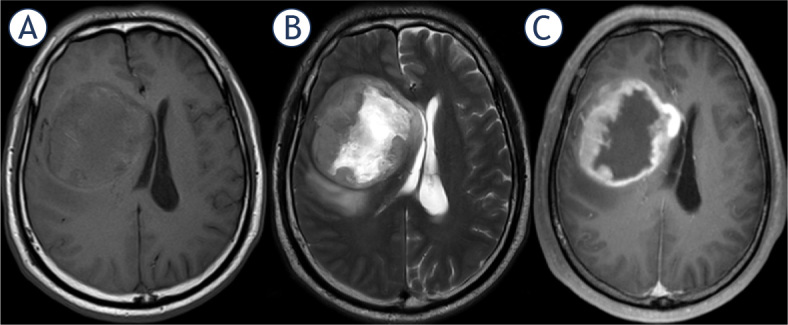
Representative MR images with imaging necrosis derived from a 53-year-old man with glioblastoma, IDH-wildtype. Shown from left to right by the order are T1WI **(A)**, T2WI **(B)**, and T1WI-CE **(C)**.

As mentioned in the introduction, examples of imaging necrosis, defined as a region within the tumor that does not enhance or shows markedly diminished enhancement, high signal intensity on T2WI, low signal intensity on T1WI, and an irregular border, are shown in Figure 2 and Supplementary Figure 2. Two experienced radiologists reviewed all conventional MRIs. Then they determined whether there was Im_necrosis_ by consensus. One of these two experienced radiologists and a third radiologist repeatedly assessed 68 cases after the initial assessment to assess the inter-observer agreement. The assessed images were randomized within each type of pathology, and the observers were blinded to the clinical and pathological information and thoroughly acquainted with the criteria.

### Pathological and molecular analysis

Pa_necrosis_ was defined according to pathological reports provided by the Pathology department of our hospital, if available. The status of *1p19q* codeletion, *EGFR* amplification, chr7 gain/10 loss (+7/−10), and *CDKN2A/B* homozygous deletion were determined by fluorescence *in situ* hybridization with a specific probe. *IDH* mutation was determined by high-throughput sequencing, including *IDH1* and *IDH2* mutations. The pathological diagnosis and grading of gliomas were reassigned according to the 2021 WHO CNS classification (Supplementary Figure 3).^4,14,15^

### Statistical analysis

Statistical Analysis Data were analyzed using IBM SPSS Statistics 26 software, the SPSSAU data scientific analysis platform (https://spssau.com/), and the R programming language (version 4.1.2, The R Foundation for Statistical Computing). Normally distributed continuous variables were compared using unpaired t-tests, whereas non-parametric tests were used for non-normally distributed variables. Descriptive data are expressed as mean ± SD, except where otherwise stated. Unpaired t-tests, non-parametric tests, and chi-squared tests were used to compare differences between parameters. Receiver Operating Characteristics (ROC) curves were used to evaluate diagnostic efficacy. Simple kappa was calculated to assess the consistency of different diagnoses and inter-observer agreement. Kaplan–Meier survival analysis was used to analyze survival data. Hazard ratios (HR) were estimated according to the Cox proportional hazard method. A two-sided *p* value < 0.05 was considered significant. Detailed statistical methods are shown in Supplementary Appendix 3.

## Results

### Patients’ demographic and clinical findings

We initially identified 150 eligible patients (median age = 46 years, range 21–79 years), and 104 (69.33%) were male (Table 1). All the diagnoses assigned to the patients according to the latest integrated histomolecular classification criterion were presented in Supplementary Figure 3 and Supplementary Table 1. Pa_necrosis_ was identified in 70/76 of highgrade gliomas (HGGs, CNS WHO grade 4) and 3/43 of low-grade gliomas (LGGs, CNS WHO grade 2 and 3) which were oligodendrogliomas, IDHmutant and 1p/19q-deleted, while Im_necrosis_ was identified in 70/76 of HGGs and 12/43 of LGGs.

**TABLE 1.** Participant demographic findings

**Parameters**	**Type**	**Imaging necrosis**		**Sum**	**t/χ^2^b**	***p***
		**Negative n (%)**	**Positive n (%)**			
Age (n = 150)	-	40.54±11.08 (n = 54)	50.39±12.47 (n = 96)	-	−4.829&	p < 0.001
Sex (n = 150)	male	36(66.67)	68(70.83)	104	0.282	0.595
	female	18(33.33)	28(29.17)	46		
*IDH* (n = 144)	wildtype	17(32.08)	69(75.82)	86	26.649	p < 0.001
	mutant	36(67.92)	22(24.18)	58		
*1p19q* (n = 109)	non-codeletion	23(51.11)	55(85.94)	78	15.746	p < 0.001
	codeletion	22(48.89)	9(14.06)	31		
*CDKN2A/B* homozygous deletion (n = 63)	non-deletion	38(100.00)	20(80.00)	58	5.745b	0.017*
	deletion	0(0.00)	5(20.00)	5		
*EGFR* amplification (n = 81)	non-amplification	8(66.67)	45(65.22)	53	0.054a	0.817
	amplification	4(33.33)	24(34.78)	28		
chr7 gain/10 loss (n = 26)	negative	10(83.33)	13(92.86)	23	0.552b	0.457
	positive	2(16.67)	1(7.14)	3		
Grade (n = 119)	high-grade	6(16.22)	70(85.37)	76	52.828	p < 0.001
	low-grade	31(83.78)	12(14.63)	43		
WHO grade (n = 119)	WHO grade 2	26(70.27)	4(4.88)	30	62.664a	p < 0.001
	WHO grade 3	5(13.51)	8(9.76)	13		
	WHO grade 4	6(16.22)	70(85.37)	76		
Integrated histo-molecular diagnoses (n = 116)	Oligodendroglioma, IDH-mutant and 1p/19q-deleted	17(45.95)	7(8.86)	24	41.238	p < 0.001
	Astrocytoma, IDH-mutant	15(40.54)	12(15.19)	27		
	Glioblastoma, IDH-wildtype	5(13.51)	60(75.95)	65		

& = Student's t statistic in this cell, and other cells in the same column represent Chi-square values. a and b = chi-square tests with continuity correction and Fisher's exact tests, respectively;

* = p < 0.05

There was 1/77 HGG without enhancement but with positive status of *EGFR* amplification, thus diagnosed as glioblastomas, IDH-wildtype, while there were 32/45 LGGs with enhancement diagnosed as oligodendrogliomas, IDH-mutant and 1p/19q-deleted (n = 15) and astrocytoma, IDH-mutant (n = 17). And most HGGs were manifested as ring enhancement, and most LGGs had patchy and punctate enhancement. All the clinical information was presented in Table 1 and Supplementary Appendix 4.

### Interobserver agreement of imaging necrosis and correlation between imaging and pathological necrosis

In this study, the following four groups were determined: Im+Pa_necrosis_ group (representing patients with both Im_necrosis_ and Pa_necrosis_, n = 74), no_necrosis_ group (representing patients without Im_necrosis_ nor Pa_necrosis_, n = 28), *Only* Im_necrosis_ group (representing patients with Im_necrosis_ but without Pa_necrosis_, n = 7), and *Only* Pa_necrosis_ group (representing patients with Pa_necrosis_ but without Im_necrosis_, n = 4) groups. Detailed clinical, imaging and paychological information of *Only* Im_necrosis_ group and *Only* Pa_necrosis_ group were shown in Table 2. We found strong agreement between Im_necrosis_ and Pa_necrosis_ (Kappa = 0.767, p < 0.001, 95%CI: 0.637–0.897).

**TABLE 2.** Detailed clinical, imaging and pathological information of Only Im_necrosis_ group and Only Pa_necrosis_ group

**Group**	**Grade**	**Sex**	**Age**	**OS (month)**	***IDH* (0:wild; 1:mutant)**	***1p19q* (0:non-codeletion; 1:codeletion)**	***CDKN2A/B* (0:non-deletion; 1:deletion)**	***EGFR* amplification (0:non-amplification; 1:amplification)**	**chr7 gain/10 loss (0:negative; 1:positive)**	**Pathology**
*Only* Pa_necrosis_ group	WHO CNS grade 4	female	63	2.5	1	0	0	NA	NA	Astrocytoma, IDH-mutant
*Only* Pa_necrosis_ group	CNS WHO grade 4	female	55	20	0	0	NA	0	NA	Glioblastoma, IDH-wildtype
*Only* Pa_necrosis_ group	CNS WHO grade 2	female	36	NA	1	1	0	NA	NA	Oligodendroglioma, IDH-mutant and 1p/19q-deleted
*Only* Pa_necrosis_ group	NA	female	34	NA	1	NA	NA	NA	NA	IDH-mutation, NOS
*Only* Im_necrosis_ group	CNS WHO grade 4	male	64	5	0	0	NA	1	NA	Glioblastoma, IDH-wildtype
*Only* Im_necrosis_ group	CNS WHO grade 2	male	40	25	1	0	0	NA	NA	Astrocytoma, IDH-mutant
*Only* Im_necrosis_ group	CNS WHO grade 3	female	55	60.06	1	1	0	NA	NA	Oligodendroglioma, IDH-mutant and 1p/19q-deleted
*Only* Im_necrosis_ group	CNS WHO grade 2	male	26	5.39	1	0	0	0	0	Astrocytoma, IDH-mutant
*Only* Im_necrosis_ group	NQ	male	40	7.19	0	0	NA	0	NA	IDH-wildtype, NOS
*Only* Im_necrosis_ group	NA	male	28	19.68	0	0	NA	0	0	IDH-wildtype, NOS
*Only* Im_necrosis_ group	CNS WHO grade 3	male	26	34.42	1	0	0	NA	NA	Astrocytoma, IDH-mutant

**FIGURE 3.**
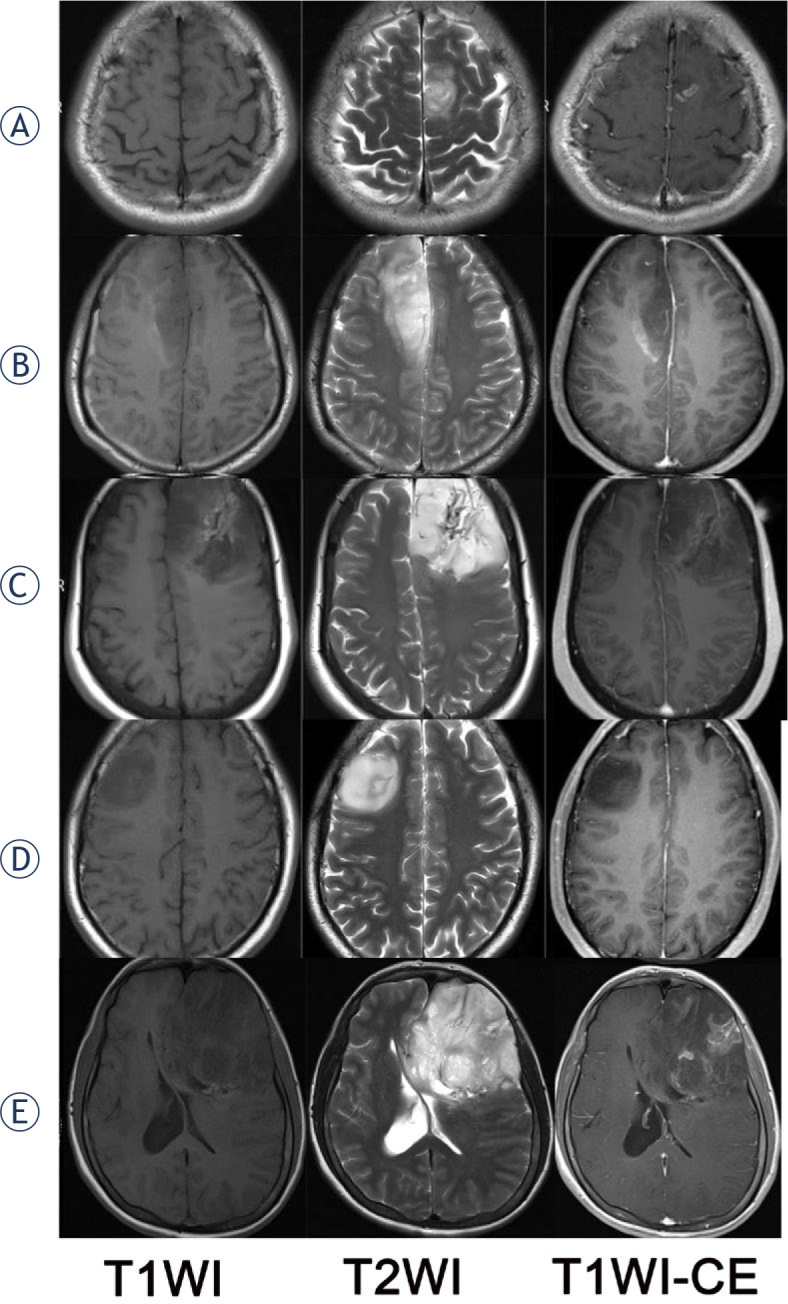
Some representative MRI images without imaging necrosis which was exactly confused in diagnosing imaging necrosis. Shown from left to right by the order are T1WI, T2WI, and T1WI-CE. **(A)** a 24-year-old man with an oligodendroglioma, IDH-mutant and 1p/19q-deleted, CNS WHO grade 2; **(B)** a 39-year-old man with an oligodendroglioma, IDH-mutant and 1p/19q-deleted, CNS WHO grade 2; **(C)** a 55-year-old woman with an oligodendroglioma, IDHmutant and 1p/19q-deleted, CNS WHO grade 2; **(D)** a 45-year-old man with an astrocytoma, CNS IDH-mutant, WHO grade 2; **(E)** a 36-year-old woman with an oligodendroglioma, IDH-mutant and 1p/19q-deleted, CNS WHO grade 2. In this case **(E)**, it showed multiple long tubular and filiform enhancement and there were some tumor areas with remarked decrease of reinforcement. But these areas are hyperintense, not hypointense, on the T1-weighted image. Comparing with CT images (not provided), calcification on these areas were just observed. So, there was no imaging necrosis in these conditions.

Besides, there was strong inter-observer agreement in identifying imaging necrosis (Kappa = 0.668, p < 0.001, 95%CI: 0.489–0.846). And the spotlike, dotted, long-strip, long tubular, and fissural enhancements (Figure 3) which were easily misdiagnosed as imaging necrosis should be avoided.

### Association of imging necrosis with integrated glioma grading

Most HGGs (85.37%) were found to have Im_necrosis_, while most LGGs (83.78%) were without Im_necrosis_. There were 4/30 WHO grade 2 patients with Im_necrosis_. Of those, two were diagnosed as oligodendrogliomas, IDH-mutant and 1p/19q-deleted, and two as astrocytomas, IDH-mutant.

Significant differences in the presence of Im_necrosis_ with a large effect size were found between HGGs and LGGs and among different grades of gliomas (Table 1, *p* < 0.001). Cochran–Armitage tests showed an upward trend in Im_necrosis_ from lower to higher grades of gliomas (*p* < 0.001). Multiple comparisons with Bonferroni correction showed that the difference between WHO grades (any two grades) and Im_necrosis_ was significant (all *p* < 0.01).

### Association of imaging necrosis and molecular profiles of gliomas

There were significant correlations between the expression of other molecular markers such as *IDH*, *1p19q*, and *CDKN2A/B* and the presence of Im_necrosis_. According to Table 1, the proportion of *IDH*-wildtype, *1p19q*-non-codeletion, or *CDKN2A/B*-positive cases with Im_necrosis_ was significantly higher than that of cases without Im_necrosis_ with a medium effect size, respectively (75.82% *vs.* 32.08%, 85.94% *vs.* 51.11%, 20% *vs.* 0, respectively). However, no significant correlation was found between Im_necrosis_ and *EGFR* amplification or +7/−10 cytogenetic signature (*p* > 0.05) (Table 1).

### Association of imaging necrosis with patient prognosis

**FIGURE 4.**
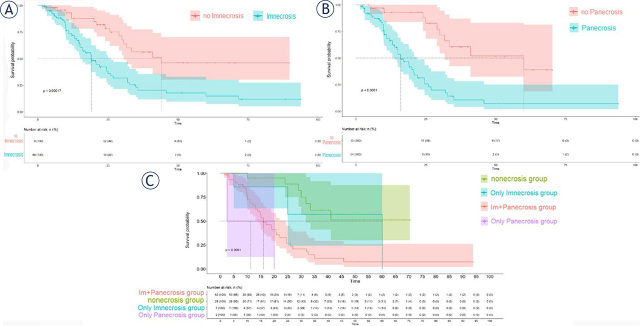
Survival curves for cases of imaging necrosis **(A)**, cases of pathological necrosis **(B),** and cases of both pathological and imaging necrosis **(C)**.

One-hundred and thirty patients were included in the final survival analysis. Compared with gliomas with Im_necrosis_, patients without Im_necrosis_ had a significantly longer survival time (*p* < 0.001, Figure 4A). By reference to the gliomas with Pa_necrosis_, patients without Pa_necrosis_ had a significantly longer survival time as well (*p* < 0.001, Figure 4B).

The differences among the OS of Im+Pa_necrosis_, no_necrosis_, *Only* Im_necrosis_, and *Only* Pa_necrosis_ groups were statistically significant (*p* < 0.01, Figure 4C). Further, after Bonferroni correction, there were significant differences between Im+Pa_necrosis_ and no_necrosis_ groups (*p* < 0.001). According to Figure 4C, the OS of the *Only* Pa_necrosis_ group (*n* = 2) was shorter than the OS of no_necrosis_ group (n = 28) and *Only* Im_necrosis_ group (n = 7). Between the two survival curves of no_necrosis_ and the *Only* Im_necrosis_ groups, there were marked crossovers, but within a certain period (time spanning about from 5-month to 40-month postoperatively), the OS of the *Only* Im_necrosis_ group was shorter than the OS of no_necrosis_ group.

Further, when added significant variables such as age, *IDH*, *1p19q*, and Im_necrosis_ into the multivariate Cox proportional hazards regression analyses, only Imnecrosis (HR = 2.113, 95% CI: 1.015–4.402, *p* = 0.046) was significant and independently related to the patients’outcome, indicating that Im_necrosis_ is an independent and unfavourable prognostic factor.

### Correlation of tumor necrosis and DCE-MRI metrics

Since pathology is the golden standard for necrosis diagnosis, we analyzed the associations with Pa_necrosis_ and DCE-MRI metrics. Most DCE-MRI metrics demonstrated a significant difference in identifying gliomas with Pa_necrosis_ with a very large effect size (Table 3). *Kep* was significantly higher for gliomas with Pa_necrosis_ than those without Pa_necrosis_, while other DCE-MRI metrics showed the opposite trend. ROCs analysis showed that the Tumor-*ve*-Mean displayed the best diagnostic performance with the largest AUC of 0.891 (95%CI: 0.788–0.995, *p* < 0.0001), and the optimal cut-off point was 0.17 with a sensitivity of 96% and specificity of 83.3%.

**TABLE 3.** Representative results of non-parametric tests and ROC analyses between DCE-related data for gliomas with or without pathological necrosis/imaging necrosis

**Parameter**	***p***	**AUC (95% CI)**	**Sensitivity**	**Specificity**	**Cut-off**
Pa_necrosis_					
Tumor-*ktrans*-Mean	< 0.001	0.824 (0.711 ~0.936)	0.94	0.625	0.07
Edema-*ktrans*-Mean	0.031*	0.655 (0.527 ~ 0.783)	0.833	0.46	0.03
Tumor-*ve*-Mean	< 0.001	0.891 (0.788 ~ 0.995)	0.96	0.833	0.17
Edema-*ve*-Mean	0.002**	0.728 (0.613 ~ 0.842)	0.34	1	0.16
Tumor-*kep*-Mean	< 0.001	0.872 (0.761 ~ 0.983)	0.833	0.86	2.48
Tumor-*iauc*-Mean	< 0.001	0.899 (0.803 ~ 0.996)	1	0.75	0.07
Im_necrosis_					
Tumor-*ktrans-*Mean	< 0.001	0.856 (0.772 ~ 0.939)	0.877	0.757	0.08
Tumor-*ve*-Mean	< 0.001	0.929 (0.872 ~ 0.986)	0.892	0.919	0.17
Edema-*ve*-Mean	0.005**	0.667 (0.558 ~ 0.776)	0.708	0.595	0.06
Tumor-*kep*-Mean	< 0.001	0.914 (0.857 ~ 0.971)	0.946	0.831	2.74
Tumor-*iauc*-Mean	< 0.001	0.909 (0.844 ~ 0.974)	0.8	0.946	0.13

* = p < 0.05;

** = p < 0.01

Similarly, we performed the analysis regarding Im_necrosis_ (Table 3), and the Tumor-*ve*-Mean displayed the best diagnostic performance as well, with the most significant AUC of 0.929 (95%CI: 0.872–0.986, p < 0.0001) and the optimal cut-off point was 0.17 with a sensitivity of 89.2% and specificity of 91.9%.

## Discussion

In this study, we investigated the clinical implication of imaging necrosis in the preoperative evaluation of glioma. We found strong agreement between Im_necrosis_ and Pa_necrosis_. Moreover, Im_necrosis_ was found to be significantly related to gliomarelated key gene mutations, such as *1p19q* noncodeletion and *CDKN2A/B* homozygous deletion. And it is an independent imaging marker for predicting tumor prognosis. Additionally, tumor parenchyma *ve* derived from DCE-MRI can help to predict tumor necrosis with high specificity.

Our study indicated strong agreement between the inter-observer agreement of Im_necrosis_ and Pa_necrosis_. And during the analysis, we found that the regions with an absence or marked decrease of enhancement inside the intensified areas were easily mistaken as Im_necrosis_. While considering pathological samples were partial, imaging observation can capture full tumors. There was a pathologically proven astrocytoma, IDH-mutant, CNS WHO grade 2, with a very short OS (5 months). We reviewed the raw data and identified that this patient had a small extent of Im_necrosis_, indicating high grade gliomas. The situation mentioned above can be avoided if a judgement of Im_necrosis_ is made, which is one unique advantage of radiographic examination. Besides, we identified seven patients with Im_necrosis_ who were diagnosed as oligodendrogliomas, IDH-mutant and 1p/19q-deleted, CNS WHO grade 2 or 3, indicating that necrosis plays a limited predictive value in oligodendrogliomas. Hence, if there is evidence of oligodendrogliomas, such as calcification and filiform or localized internal homogeneous enhancement, Im_necrosis_ does not indicate a high-grade tumor. Besides, Waqar *et al.* reported this kind of reinforcement as a “chicken wire” appearance with the explanation that oligodendroglioma vasculature often was described as a network of regular fine branching capillaries.^16,17^

Previous studies have highlighted that Im_necrosis_ is an independent unfavorable prognosis factor. ^5,6,10,18,19,20^ Our results were in accordance with their findings. Besides, the latest WHO CNS classification emphasizes the role of molecular markers, such as *IDH*, *1p19q*, *CDKN2A/B*, 7+/10−, and *EGFR*, in the diagnosis and prediction of the prognosis of gliomas.^4^ From this prospect, Im_necrosis_ might be more critical than Pa_necrosis_ since it can be non-invasively obtained before operation. However, there was no significant difference between the expressions of 7+/10− cytogenetic signature or *EGFR* amplification and the presence of Im_necrosis_. This negative result might be due to the small sample size and insufficient number of events.

In this study, we also sought quantitative metrics for indicating tumor necrosis. Our results revealed that, compared with tumor without Im_necrosis_/Pa_necrosis_, DCE-derived metrics in tumor parenchyma, except *kep*, were significantly higher in gliomas with Im_necrosis_/Pa_necrosis_. And *ve* in tumor parenchyma demonstrated the highest diagnostic efficiency in identifying tumor necrosis with high sensitivity and specificity. Significantly high DCE-MRI metrics may be attributed to gliomas growing uncontrollably fast, resulting in severe hypoxia and necrosis. Thus, an extensively hyperpermeable vasculature is generated, resulting in inadequate oxygen and supplements delivery. The greater the levels of perfusion and permeability in the tumor tissue, the higher the *ktrans* and *ve* and the higher the degree of tumor malignancy.^21,22,23^ Hence, DCE-MRI metrics, especially *ve* in tumor parenchyma (cut-off value: 0.17), might be a supplementary metric to the morphological observation for delineating tumor necrosis.

The current study has some limitations. First, since evidence of pathological necrosis was obtained from pathology reports of the same hospital, there may be an observation bias. However, this study, based on clinical real-world evidence, can exactly address the current clinical deficits. Second, this is a single-center study; subgroups analysis had a small sample, which might result in insufficient power to reach definite conclusions. Further multicenter studies with large sample sizes will help improve the efficacy of Im_necrosis_ in predicting the expression of molecular markers and prognosis.

## Conclusions

Based on the latest WHO CNS guidelines, the present study depicted the importance of imaging necrosis in diagnosing gliomas. Detection of imaging necrosis in gliomas probably suggests an HGG unless there is imaging evidence for oligodendrogliomas, IDH-mutant and 1p/19q-deleted. Imaging necrosis was significantly associated with glioma-related key gene mutations, such as *1p19q* non-codeletion and *CDKN2A/B* homozygous deletion. And it is an independent imaging marker for predicting tumor prognosis. Additionally, the Tumor-*ve*-Mean derived from DCE-MRI can help to predict necrosis with high sensitivity and specificity. Overall, in this study, we re-evaluated the imaging necrosis in the assessment of gliomas and provided a feasible solution to solve the frequent diagnostic dilemma of gliomas.

## Supplementary Material

Supplementary Material Details
