# Supplementary material for: Looking through the imaging perspective: the importance of imaging necrosis in glioma diagnosis and prognostic prediction – single centre experience
Source: Radiol Oncol. 2024 Feb 21;58(1):23–32. doi: 10.2478/raon-2024-0014 (PMC10878771; doi:10.2478/raon-2024-0014)
Supplement: Supplementary file 1 — Supplementary Material Details [file raon-2024-0014-sm.pdf]

Looking through the imaging perspective: the importance of imaging necrosis in glioma diagnosis and prognostic prediction – single centre experience

Hui Ma, Shanmei Zeng, Dingxiang Xie, Wenting Zeng, Yingqian Huang, Liwei Mazu, Nengjin Zhu, Zhiyun Yang, Jianping Chu, Jing Zhao

Radiol Oncol 2024; 58(1): 23-32.

doi: 10.2478/raon-2024-0014

APPENDIX 1. MRI parameters

Participants underwent conventional and dynamic contrast-enhanced MR perfusion imaging (DCE-MRI) using a 3T MR system (Magnetom Verio, Siemens Medical Solutions, Erlangen, Germany) with a 64-channel head-neck coil. The parameters of the conventional MRI were as follows: transversal T2WI (repetition time [TR], 4200 ms; echo time [TE], 109 ms; field of view [FOV], 243 mm × 194 mm; slice thickness, 6 mm; voxel resolution, 0.6 mm × 0.6 mm× 6.0 mm) and transversal T1WI (TR, 2000 ms; TE, 17 ms; FOV, 230 mm × 187 mm; slice thickness, 6.0 mm; voxel resolution, 0.7 mm × 0.7 mm × 6.0 mm) were obtained. For, DCE-MRI, T1-VIBE was applied at two different flip angles (2° and 15°) to calculate the T1 maps, and it had the following parameters: TR, 3.83 ms; TE, 1.37 ms; slice thickness, 3.5 mm; FOV, 220 mm × 220 mm; voxel resolution, 1.4 mm × 1.4 mm × 3.5 mm. DCE-MRI was acquired with time-resolved angiography using stochastic trajectories (TWIST) sequence; the parameters were the following: TR, 4.89 ms; TE, 1.88 ms; flip angle, 12°; slice thickness, 3.5 mm; for each measurement, 4.77 s; FOV, 220 mm × 220 mm; 75 measurements, total scan time of 358 s; voxel resolution, 1.4 mm × 1.4 mm × 3.5 mm; contrast media (0.2 mmol/kg body weight of Gd DTPA, Magnevist, Schering, Berlin, Germany); contrast median injection rate, 4 mL/s, followed by 20 mL of 0.9% saline flush using the same injection speed. The infusion was started from the fifth measurement. Post-contrast 3D T1mprage sagittal T1-weighted images (TR, 2300 ms; TE, 2.43 ms; section thickness, 0.75 mm; FOV, 240 mm × 225 mm; voxel resolution, 0.8 mm × 0.8 mm × 0.8 mm) were obtained after DCE-MRI.

APPENDIX 2. Supplementary image processing methods

The analysis of DCE-MRI data was performed using a commercial software tool (TISSUE 4D; Siemens Healthcare, Erlangen, Germany). The value for the arterial input function was automatically calculated using the software. TISSUE 4D was based on the two-compartment model.

APPENDIX 3. Detailed statistical methods

Distribution of data was assessed on histograms and with Shapiro–Wilk tests. Categorical variables were appropriately compared using Pearson chi-squared tests, chi-squared tests with continuity correction, or Fisher's exact tests. Cochran-Armitage tests were used to evaluate the trends of glioma grades. Receiver Operating Characteristics (ROC) curves were used to evaluate diagnostic efficacy. For continuous dependent variables, Cohen's d was used as a measure of effect size (small effect = 0.2, medium effect = 0.5, large effect = 0.8). For categorical variables, Phi or Cramer's V coefficients were used as effect size indicators (small effect = 0.1, medium effect = 0.3, large effect = 0.5). Simple kappa was calculated to assess the consistency of different diagnoses and interobserver agreement. Kappa values were interpreted using the following criteria: < 0.20 = poor agreement; 0.21–0.40 = general agreement; 0.41–0.60 = moderate agreement; 0.61–0.80 = strong agreement; and 0.81–1.00 = excellent agreement. Survival data was analyzed by Kaplan–Meier survival analysis using the R "survival" and "survminer" packages, and differences in survival were evaluated with the log-rank tests. Multivariable analyses with the Cox proportional-hazards model were used to estimate the simultaneous effects of prognostic factors on survival using stepwise forward likelihood-ratio testing. Hazard ratios (HR) were estimated according to the Cox proportional hazard method. For multiple comparisons, the p-value was set at the Bonferroni-corrected significance level.

APPENDIX 4. Extra detailed patients' demographic and clinical findings

One hundred and thirteen people were finally included who demonstrated to be with both imaging and pathological necrosis. Ninety-nine patients were included in the final survival analysis of groups with or without pathological necrosis. Among the 102 (68%) patients who underwent DCE-MRI, there were 24 cases without pathological necrosis, 50 with pathological necrosis, 37 without imaging necrosis, and 65 with imaging necrosis. For the follow-up, 20 (13.33%) patients were lost-to-follow-up, 66 (44%) patients had died, and 64 (42.67%) patients were alive.

SUPPLEMENTARY TABLE 1. The final diagnoses assigned to the included patients

Integrated diagnoses	CNS WHO grade			sum
	WHO grade 2	WHO grade 3	WHO grade 4	
Astrocytoma, IDH-mutant	14	5	8	27
Oligodendroglioma, IDH-mutant and 1p/19q-deleted	16	8	0	24
Glioblastoma, IDH-wildtype	0	0	65	65
IDH-mutation, NOS	-	-	-	7
IDH-wildtype, NOS	-	-	-	21
Not diagnosed	-	-	3	6

"-" represents the numbers are uncertain; "not diagnosed" means that cases could not be reclassified because of unknown IDH status, including 3 ones of CNS WHO grade 4 with 1p19q non-codeletion and pathological necrosis, and could only be identified morphologically as adult diffuse glioma; NOS = not otherwise specified. it represents a lack of necessary molecular or histological diagnostic information for integrated diagnosis, but the status of gene IDH was known.

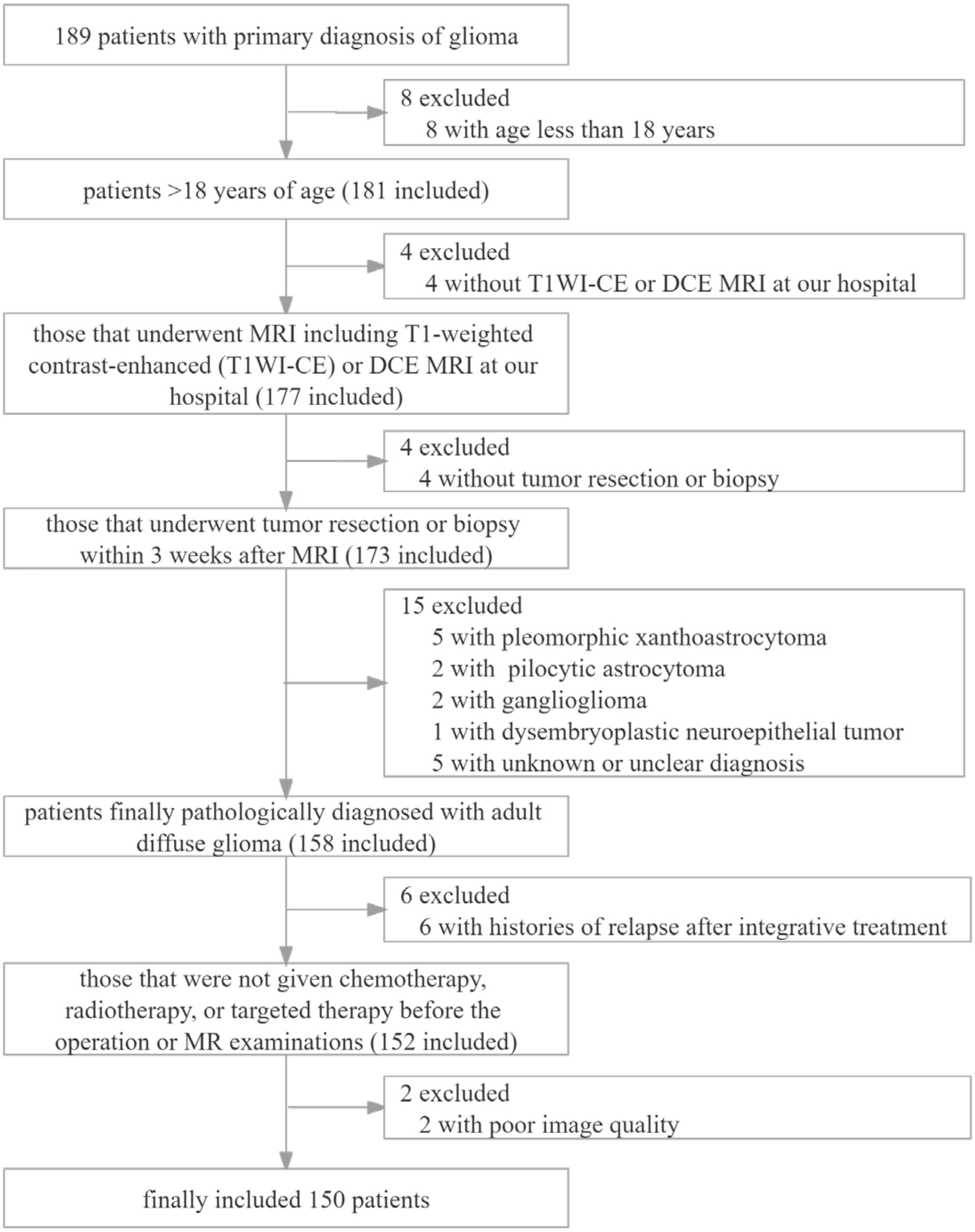

SUPPLEMENTARY FIGURE 1. Description of the inclusion and exclusion criteria.

T1WI-CE = contrast-enhanced T1-weighted image

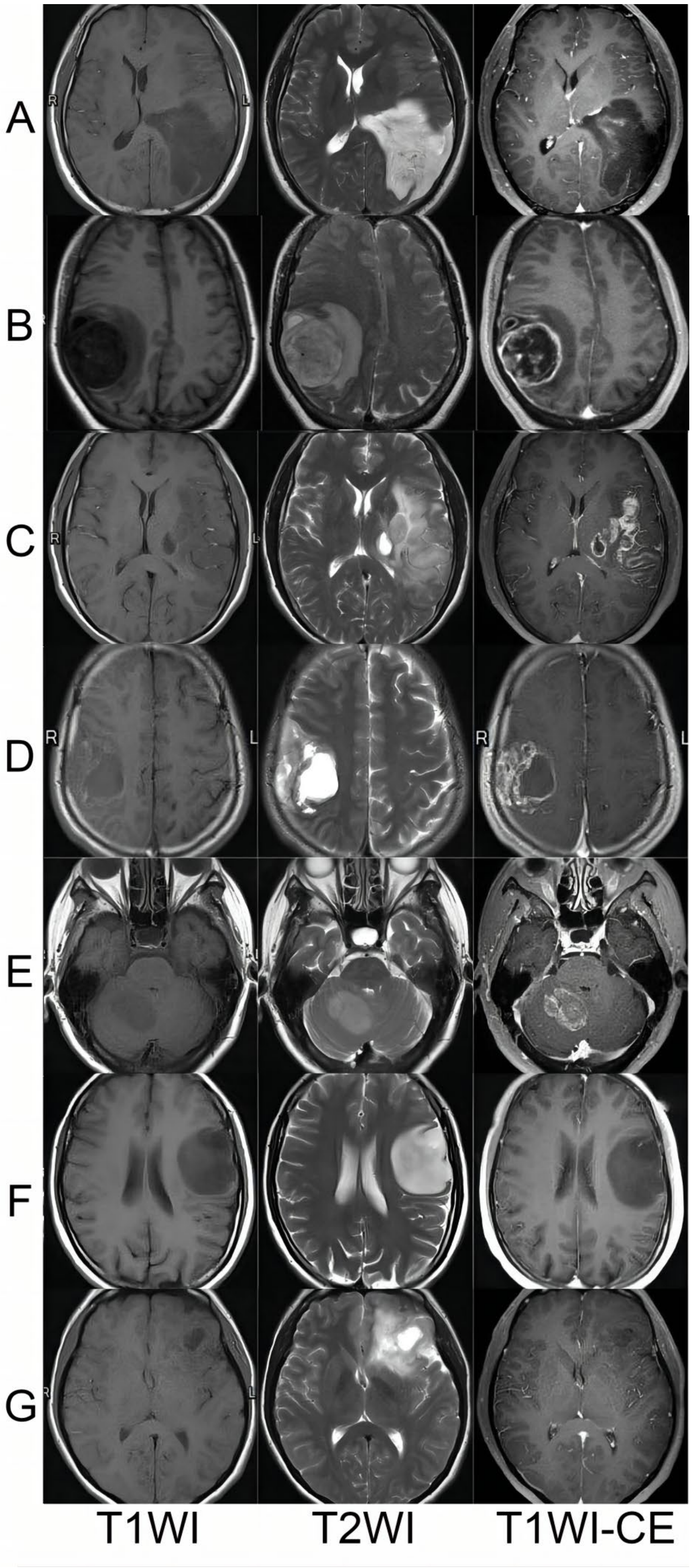

SUPPLEMENTARY FIGURE 2. The representative MR images with (**A-D**) or without (**E-G**) imaging necrosis. Shown from left to right by the order are T1WI, T2WI, and T1WI-CE = contrast-enhanced T1-weighted image. (**A**) a 40-year-old man with an astrocytoma, IDH-mutant, CNS WHO grade 2; (**B**) a 53-year-old woman with glioblastoma, IDH-wildtype, CNS WHO grade 4; (**C**) a 51-year-old man with an oligodendroglioma, IDH-mutant and 1p/19q-deleted, CNS WHO grade 2; (**D**) a 36-year-old man with an oligodendroglioma, IDH-mutant and 1p/19q-deleted, CNS WHO grade 3; (**E**) a 63-year-old woman with an astrocytoma, IDH-mutant, CNS WHO grade 4; (**F**) a 31-year-old woman with an astrocytoma, IDH-mutant, CNS WHO grade 2; (**G**) a 29-year-old woman with an oligodendroglioma, IDH-mutant and 1p/19q-deleted, CNS WHO grade 2.

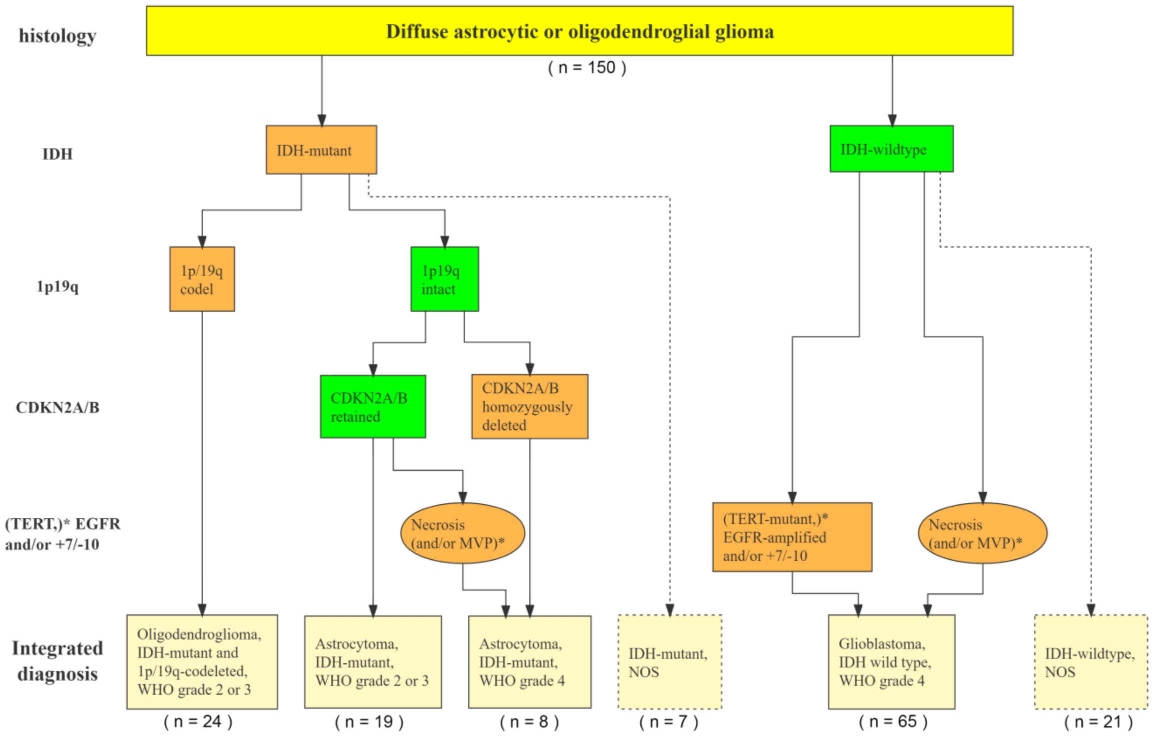

SUPPLEMENTARY FIGURE 3. Diagnostic algorithm for the integrated classification of adult-type diffuse glioma according to the latest WHO classification edition.

*not certain in this study due to some limitation of technology; n = indicates the final number of cases included in the study
